# Oil of Turpentine as an Excitant of Uterine Contraction

**Published:** 1854-01

**Authors:** 


*Oil of Turpentine as an Excitant of Uterine Contraction.*—Dr. C. Archer, of Va., recommends emenata of oil of turpentine, (℥j in gruel, q. s.) to reproduce labor pains which have subsided, or to strengthen those which are inefficient. Two reasons present themselves in favor of this remedy—its safety and the ease with which it can always be obtained.—*From the N. H. Journal of Medicine.*

